# Cross-Kingdom Similarities in Microbiome Ecology and Biocontrol of Pathogens

**DOI:** 10.3389/fmicb.2015.01311

**Published:** 2015-11-25

**Authors:** Gabriele Berg, Robert Krause, Rodrigo Mendes

**Affiliations:** ^1^Institute of Environmental Biotechnology, Graz University of Technology and ACIB Austrian Centre of Industrial BiotechnologyGraz, Austria; ^2^Department of Internal Medicine, Medical University of GrazGraz, Austria; ^3^Laboratory of Environmental Microbiology, Embrapa EnvironmentJaguariuna, Brazil

**Keywords:** microbiome, biocontrol agent, rhizsophere, pathogens, ecological theories

“Imagination is more important than knowledge.”

-Albert Einstein

## Introduction

The concept of “gut and root microbiota commonalities” was already presented by Ramírez-Puebla et al. (2013); they discussed a lot of similar functional traits, host-bacteria interactions as well as evolutionary trends but also several differences. Based on deeper insights obtained by omics technologies, Mendes and Raaijmakers (2015) recently presented their concept that the structure and function of rhizosphere and gut microbiomes show cross-kingdom similarities. In parallel, Hacquard et al. (2015) analyzed similarities of the microbiota composition across plant and animal kingdoms and found only little overlap comparing fish gut and plant root communities. They explained the differences by various start inoculants and abiotic, niche-specific factors. In this context, to establish concepts is pivotal in microbial ecology for the critical evaluation of the immense amount of data obtained by omics technologies, not only for conceptual work in microbial ecological theories (Prosser et al., 2007), but also for translational fields such as biocontrol of pathogens (Berg et al., 2013). Therefore, we would like to extend the concept of “cross-kingdom similarities” presented by Mendes and Raaijmakers (2015) to an ecological context, which is shared for host-associated microbiomes beyond the boundaries of their respective kingdoms. Finally, we discuss the impact and implications of microbiome ecology on biocontrol of pathogens in plants and in humans.

## Similarities in host-associated microbiome ecology

1. **Each host provides microhabitats with different abiotic conditions, which shape the structure of microbial communities. Despite their specific composition, communities are connected to each other and share microbial populations**. The different microhabitats of plants carrying their individual names, e.g., rhizosphere, phyllosphere, endosphere, have been well-studied for decades (Philippot et al., 2013; Berg et al., 2015; Hardoim et al., 2015). In parallel, human microenvironments and their specific microbiomes have been thoroughly studied over the past years (Blaser et al., 2013). In addition, microbial exchanges between different host's compartments or niches were analyzed. For plants, the microbial transfer from soil to the rhizo- and endo-sphere was analyzed in particular (Edwards et al., 2015). In humans, analysis of dent-associated microbial communities was shown to be important for the health of the whole body including the placenta during pregnancy periods (Aagaard et al., 2014).
2. **There is a co-evolution between the host and its microbiome**. According to the hologenome theory of evolution, hosts and their associated microbiomes can be viewed as a superorganism, where microorganisms play a key role in the evolution of the host (Zilber-Rosenberg and Rosenberg, 2008). This co-evolution has already been hypothesized based on culture-dependent results obtained for the rhizosphere of ancient and modern wheat cultivars (Germida and Siciliano, 2001). Genotype-specific microbiomes, which were recently identified in maize (Bouffaud et al., 2012), barley (Bulgarelli et al., 2015), lettuce (Cardinale et al., 2015) as well as in the model plant Arabidopsis (Schlaeppi et al., 2014), support this hypothesis. Crop breeding is a strong driver of natural evolution (Pérez-Jaramillo et al., 2015). In some cases, the breeding strategy was targeted against pathogens, but historically it was mainly a random selection process for phenotypes. There is also evidence of co-evolution between humans and their microbiome (Schnorr, 2015), and the combination of both host-microbe and microbe-microbe interactions has likely shaped host-associated microbial communities and selected for beneficial interactions.
3. **Vertical transmission of a core microbiome plays a role in the maintenance of functional diversity**. In humans, vertical transmission of microbes takes place during the birth process, breast feeding, and close contact between mother and new-born; the transmission of microbes during this phase of life was identified as essential for the prevention of chronic diseases in later life (Blaser, 2014). For plants, the picture is more differentiated within the individual phylogenetical branches. For example, plant phyla such as orchids (*Orchidaceae*) or bryophytes need specific microorganisms for germination as well as for early stage plant growth. Vertical transmission of a core microbiome was shown not only within the sporophyte of mosses but also within seeds of higher plants (Bragina et al., 2012; Berg et al., 2015). Although the seed microbiome has been explored mainly through culture-based studies and therefore remains largely unknown, all seeds seem to carry complex but specific microbial communities (Barret et al., 2015; Berg pers. communication), which are transferred to the offspring.
4. **The microbiome structure varies during life cycle**. The age-dependence of microbiome structures was already mentioned by Mendes and Raaijmakers (2015). Interestingly, the first period of life for plants and humans is characterized by “early stage pertubations” (Blaser, 2014), and this is the period where dysbiosis resulting in diseases is more frequent. During active life, healthy eucaryotic hosts are characterized as having stable host-microbe interactions. During host's life cycle, stages with important changes in metabolism are mainly accompanied by microbiome shifts (Smalla et al., 2001; Berg and Smalla, 2009). For plants, this is the flowering period, and for humans, the fertile period of woman is one such example. In the host's senescent phase, the general microbial diversity decreases, and the role of *Enterobacteriaceae* increases due to their role as degrader (Berg et al., 2015). Strikingly, members of the *Archaea* domain showed the same ecological behavior; they colonize old/senescent plants and humans in high abundances (Probst et al., 2013; Müller et al., 2015). In addition, there is an impact of physiological rhythms of the host, e.g., the activity of all organisms is regulated by diverse molecular clock mechanisms that synchronize physiological processes to diurnal environmental fluctuations. Recently, Thaiss et al. (2014) showed that the intestinal microbiota in humans exhibits diurnal oscillations that are influenced by feeding rhythms, leading to time-specific compositional and functional profiles of the microbiome over the course of a day. Ablation of host molecular clock components or induction of jet lag leads to aberrant diurnal microbiota fluctuations and dysbiosis, driven by impaired feeding rhythmicity. Plants, as photosynthetically active organisms, show strong daily rhythms, influencing the release of root exudates. We therefore assume that this rhythm in turn affects the rhizosphere microbiome.
5. **Most host-associated microbiomes represent an interplay of *Bacteria***, ***Archaea* as well as eukaryotic microorganisms**. A high taxonomic diversity can be observed in the published metagenomes studies; *Bacteria* are often the most abundant microorganisms, i.e., cell counts, in the microbiomes and act as a key player for the host functioning (keystone species). *Archaea*, formerly considered to be inhabitants of extreme environments, were also found to be host-associated, e.g., in the human gut, on human skin as well as in the endosphere of plants (Probst et al., 2013; Berg et al., 2015). Less is known about the functional interplay between individual different microbial kingdoms. Bacteria-fungi networking were shown to be very important (Frey-Klett et al., 2011), however the function of *Archaea* in relation to eukaryotic hosts is yet not understood.
6. **Functional diversity within a microbiome is more important than structural diversity**. Although structural diversity was identified as paramount to the prevention avoidance of pathogen invasion/outbreaks (van Elsas et al., 2012), we also learned that the individual component is important for the microbiome structure and, finally, the functional diversity is crucial (Chapelle et al., 2015). In accordance, Mendes et al. (2013) proposed that studies aiming to use microbial consortia as strategy to promote plant growth and health should assemble beneficial core microbiomes more from a functional perspective than based on taxonomic classification only.
7. **Human interventions cause a loss of associated microbial diversity**. The relationship between the loss of functionally conserved ancient microbial inhabitants and the current metabolic disease and allergies of humans is stressed by Blaser (2014). In plants, domestication has negatively impacted the rhizosphere microbiome assembly and functions (Pérez-Jaramillo et al., 2015). Organic agricultural systems show a higher diversity than conventionally managed systems (Berg et al., 2013). Moreover, natural vegetation including endemic plants are characterized by an impressive microbial diversity and networking, which is often reduced in agricultural systems (Köberl et al., 2011). In parallel to human diseases, it is well known that long-term agricultural monoculture potentially results in disease outbreaks, which are often followed by establishment of disease suppressive microbiomes (Mendes et al., 2011; Kwak and Weller, 2013). This was also experimentally evidenced in a field plot by Santhanam et al. (2015). In the future, hopefully biocontrol approaches can be extended to microbiome control and design strategies to prevent diseases.

## Cross-kingdom similarities in biocontrol of pathogens

The human microbiome has been predicted to become one of the most important tools for personalized health and targeted medicine (De Vrieze, 2013). In addition, to understand the plant microbiome is crucial to find solutions for environmentally friendly agriculture especially under climate change condition and a growing human population (Berg et al., 2013). Altogether, the modulation of microbiota is currently a growing area of research as it just might hold the key to treatment. Mueller and Sachs (2015) call this an engineering approach for host-mediated microbiome selection. They proposed designed microbiomes, which enhance host functions, contributing to host health and fitness. Biological control exists much longer as an environmentally sound and effective means of reducing pathogens and their symptoms through the use of natural antagonists. All ecological rules or patterns presented above have an impact on the development of novel biocontrol approaches and offer an enormous potential for biotechnology. Summarizing, a microbiome approach for biological control should consider the following: (i) the specific composition of microbiomes at different developmental stages and for different species/cultivars/ecotypes, (ii) core microbiomes as an important source for biologicals, (iii) the microbiome view should include members from the three domains of life, that is, *Bacteria, Archaea*, and *Eukaryotes*, (iv) functional diversity within a microbiome is often more important than structural diversity, and (v) the loss of diversity caused by human intervention should be compensated.

**Figure 1:**
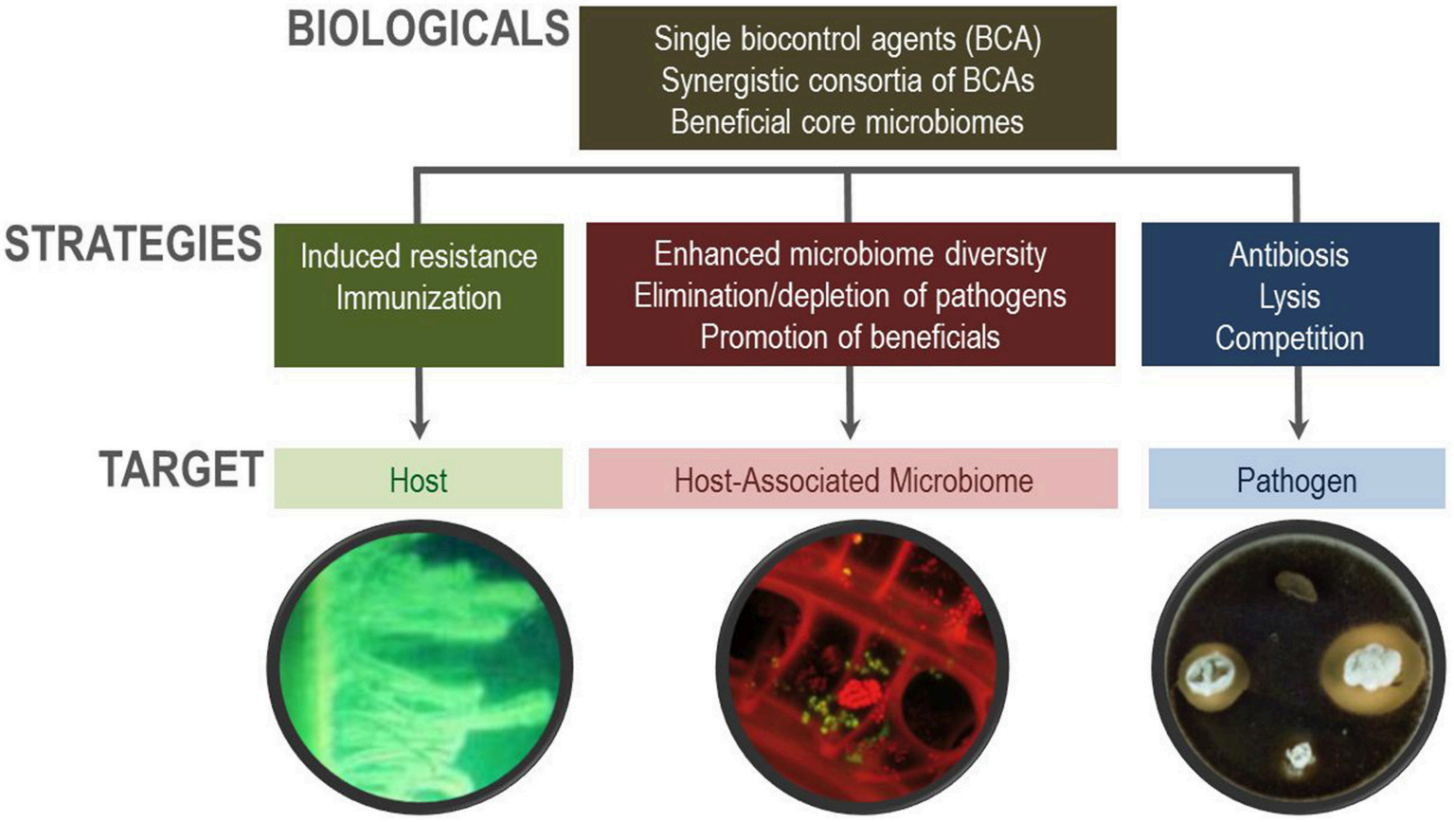
**Overview of various biocontrol strategies**.

While in the past mainly single organisms were used as biocontrol agents (BCAs) in human medicine as well as for agricultural purposes, e.g., *Bacillus* strains for plants and *Lactobacillus* strains for humans, it is now possible to develop predictable microbiome-based biocontrol strategies and to avoid inconsistent effects of the first generation of biologicals (Figure 1). These novel biocontrol strategies can not only be used to suppress pathogens, but they can also be effectively used to establish microbiomes in a desirable beneficial composition for particular purposes in the future (Berg et al., 2013). Diversity vs. pathogenicity should be an important criterion for microbiome design (van Elsas et al., 2012). This was shown by Ratner (2015) combining fecal transplants with microbial cocktails against inflammatory bowel disease. In parallel, suppressive soils were used with biologicals to supress plant diseases as Panama disease in bananas (Xue et al., 2015) and damping-off in sugar beet (Mendes et al., 2011). Stress protection agents such as *Stenotrohomonas rhizophila* are able to protect maize against drought but they also shift the whole plant-associated community and overgrow or eliminate latent fungal pathogens (Alavi et al., 2013). In addition to current developments in probiotics, prebiotics, synbiotics, and psychobiotics as defined by Wasilewski et al. (2015), many more translations are however possible, e.g., combining biologicals for plant and human health.

Functional diversity is an important aspect for health but also for biocontrol. Therefore, the mode of action plays a crucial role for biocontrol. In addition to the well-studied interaction with hosts and pathogens, the interaction with the indigenous microbiome has to be studied (Figure 1). Biologicals cause a microbiome shift, in parallel to antibiotics, but the overall goal is that they (i) enhance indigenous microbial diversity, (ii) eliminate (minor) pathogens or avoid pathogen overgrowth, and (iii) promote indigenous beneficials. Recent studies have shown promising results, including the use of biologicals in plants to enhance structural microbial diversity (Erlacher et al., 2014) as well as those applied to elderly people to improve functional diversity (Eloe-Fadrosh et al., 2015).

Altogether, there are a lot of similarities in biocontrol approaches for plants and humans. Although, single strain-based approach for biological control has begun more than 100 years ago, inconsistent control results and the fact that only a limited number of success cases exists made its use and acceptance difficult. Now we have the tools to move from a single isolate- to a community-based biocontrol approach and develop predictable biocontrol strategies on the basis of the microbiome ecology. However, there are still several hurdles in this field, including for example the formulation and shelf life of microbial communities. Furthermore, the occurrence of potential pathogens and diverse resistomes—the sum of antibiotic resistance genes—in all microbiomes needs a conceptual framework in biocontrol and microbial ecology theories.

## Author contributions

All three authors contribute to the opinion paper.

### Conflict of interest statement

The authors declare that the research was conducted in the absence of any commercial or financial relationships that could be construed as a potential conflict of interest.
